# Multimodal hippocampal and amygdala subfield volumetry in polygenic risk for Alzheimer's disease

**DOI:** 10.1016/j.neurobiolaging.2020.08.022

**Published:** 2021-02

**Authors:** Amy N. Murray, Hannah L. Chandler, Thomas M. Lancaster

**Affiliations:** a Cardiff University Brain Research Imaging Centre (CUBRIC), School of Psychology, Cardiff University, Cardiff, United Kingdom; b Dementia Research Institute at Cardiff University, School of Medicine, Cardiff University, Cardiff, United Kingdom; c School of Psychology, Bath University, Bath, United Kingdom

**Keywords:** Polygenic, Multimodal MRI, Hippocampus, Amygdala, Alzheimer's disease

## Abstract

Preclinical models of Alzheimer's disease (AD) suggest that volumetric reductions in medial temporal lobe (MTL) structures manifest before clinical onset. AD polygenic risk scores (PRSs) are further linked to reduced MTL volumes (the hippocampus/amygdala); however, the relationship between the PRS and specific subregions remains unclear. We determine the relationship between the AD-PRSs and MTL subregions in a large sample of young participants (N = 730, aged 22–35 years) using a multimodal (T1w/T2w) approach. We first demonstrate that the PRSs for the hippocampus/amygdala predict their respective volumes and specific hippocampal subregions (pFDR < 0.05). We further observe negative relationships between the AD-PRSs and whole hippocampal/amygdala volumes. Critically, we demonstrate novel associations between the AD-PRSs and specific hippocampal subfields such as CA1 (β = −0.096, pFDR = 0.045) and the fissure (β = −0.101, pFDR = 0.041). We provide evidence that the AD-PRS is linked to specific MTL subfields decades before AD onset. This may help inform preclinical models of AD risk, providing additional specificity for intervention and further insight into mechanisms by which common AD variants confer susceptibility.

## Highlights

• Polygenic risk for Alzheimer's disease (AD-PRS) explains significant proportion of AD.
• AD-PRS also linked to hippocampus and amygdala volume.
• AD-PRS is negatively associated with specific hippocampal subfields.
• Polygenic AD models help us understand genetic contributions to medial temporal lobe nuclei.

## 1 Introduction

Genome-wide association studies (GWAS) demonstrate that Alzheimer's disease (AD) is substantially heritable (58%–78%), where a substantial proportion of this variance can be explained by cumulative polygenic effects (Gatz et al., 2006). Polygenic risk scores (PRS) are a powerful approach for detecting AD risk decades before the onset of disease by combining the cumulative effect of individual single-nucleotide polymorphisms (SNPs) (Long et al., 2017) and explaining 23%–53% heritability (Lee et al., 2018; Ridge et al., 2013, 2016).

Several preliminary studies have explored the influence of individual SNPs identified via AD GWAS such as loci within *APOE*, *CLU, BIN1*, or *PICALM* on brain structure and function, broadly suggesting that common variation within these genes are associated with AD and may influence brain structure and function decades before disease onset (Chauhan et al., 2015; Ferencz et al., 2014; Sperling et al., 2011; Trachtenberg et al., 2012; Yang et al., 2016; Zhang et al., 2015). However, these variants have a modest effect in predicting AD and related pathophysiology. The PRS based on the most recent GWAS have considerably more predictive utility for AD risk (Escott-Price et al., 2015, 2017). Although AD-PRSs hold promise for understanding how to predict AD, the mechanisms by which the combined effects of the AD SNPs confer susceptibility is relatively unknown. Imaging genetics is one such approach, which may help reveal the neurobiological mechanisms by which genetic loci confer risk for AD.

Neuroimaging studies have consistently observed both global and local atrophic changes during early stages of AD in the medial temporal lobe (MTL) structures, including the amygdala, hippocampus, entorhinal cortex (ERC), and parahippocampal gyrus (Braskie et al., 2013; Jack and Holtzman, 2013; Petrella et al., 2003; Poulin et al., 2011; Serra et al., 2010; Thangavel et al., 2008). Both the hippocampus and amygdala are key subcortical nodes affected in AD-linked neurodegeneration (Hampel et al., 2014; Petrella et al., 2003; Poulin et al., 2011); however, they are not homogenous structures and comprise of a number of interconnected anatomically and functionally distinct subfields (Bocchetta et al., 2019; Saygin et al., 2017). Specific hippocampal subfields may be more susceptible to atrophy in those with AD compared with healthy controls; such as the ERC, subiculum, CA1, CA3, dentate gyrus, and CA4 (Gomez-Isla et al., 1996; Price et al., 2001; Wisse et al., 2014), suggesting that AD pathophysiology may have disproportionate influence on specific hippocampal architecture.

Combining AD-PRS and neuroimaging data may help improve AD detection strategies (Serra et al., 2010) by identifying precise markers of early AD risk (Braskie et al., 2011; Whalley et al., 2012) before the manifestation of clinical symptoms. Furthermore, recent GWAS studies show that hippocampal and amygdala volumes are also heritable (Di Paola et al., 2007; Sperling et al., 2011), making them attractive candidates to probe for AD genetic effects. These regions have also provided utility in anticipating mild cognitive impairment to AD (Braskie et al., 2013; Hampel et al., 2014; Jack and Holtzman, 2013; Petrella et al., 2003; Poulin et al., 2011; Serra et al., 2010). One preliminary study linked AD-PRS to an increased rate of volume decline in the ERC and the subiculum (Harrison et al., 2016); however, this observation was based on an AD-PRS of the GWAS loci/family history in a small sample (N = 66), so the relationship between the broad genetic architecture of AD and MTL subregions across a broad population remains largely unresolved.

In the present study, we determine the association between AD GWAS loci and specific MTL subfields and subnuclei in a large, asymptomatic sample (N = 1109, ages 22−35 years). We anticipate that AD-PRS will be negatively correlated with these subfield/subnuclei volumes based on prior studies of whole MTL volume (Biffi et al., 2010; Foley et al., 2017; Lancaster et al., 2019; Lupton et al., 2016; Mormino et al., 2016). We further aim to determine that polygenic contributions to the amygdala and hippocampus are reliably measured using recent hippocampus and amygdala GWAS as training data (Hibar et al., 2017; Satizabal et al., 2019).

## 2 Methods

### 2.1 Magnetic resonance imaging data acquisition

Data were drawn from the March 2017 public data release from the Young Human Connectome Project (YA-HCP) database. The WU-Minn HCP 1200 Subjects Release (S1200) includes 1109 healthy young adult participants’ T1 and T2 structural MR scans, available to download at https://db.humanconnectome.org/app/template/Login.vm. Participants were aged 22–35 years for all inclusion/exclusion criteria (Van Essen et al., 2013). We restricted the HCP to participants who self-reported Caucasian descent to minimize ethnicity differences between the training GWAS and the MRI data samples (see Table 1 for further demographic details). Protocol used a customized Siemens 3T “Connectome Skyra” that increases the maximum gradient strength from 40 mT/m to 100 mT/m with a 32-channel head coil and Siemens product (MPRAGE and SPACE) sequences. To acquire T1-weighed images, the protocol included slice thickness 5.0 mm, TR 2.4 ms, TE 2.14 ms, TI 1000 ms, BW 210 Hz/Px, and flip angle 8°. T2-weighed image acquisition protocol included TR 3.2 ms, TE 565 ms, BW 744 Hz/Px, and variable flip angles. Both protocols used 224 × 224 mm field of view and 0.7 mm isotropic voxel size. Scan protocol and information on the HCP pedigree/kinship structure can be found at http://www.humanconnectome.org/storage/app/media/documentation/s1200/HCP_S1200_Release_Reference_Manual.pdf.

**Table 1:** Participant demographics

Covariate	Combined sample	*APOE* ε4 (−)	*APOE* ε4 (+)	*p*
Sex (n)				0.645
Male	343	260	83	
Female	387	300	87	

	M	SD	M	SD	M	SD	
Age	29.04	3.61	28.88	3.61	29.576	3.550	0.026
Education	15.08	1.7	15.09	1.69	15.059	1.705	0.819
eTIV	1,603,258.84	176,743.26	1,600,579.0	176,620.6	1,612,086.0	177,381.6	0.458

Sex was tested using χ^2^ test. All other demographics were tested via 2-sample *t*-test.

Key: Education, number of years; eTIV, estimated total intracranial volume; M, mean; SD, standard deviation.

### 2.2 Structural MRI preprocessing

**Fig. 1:**
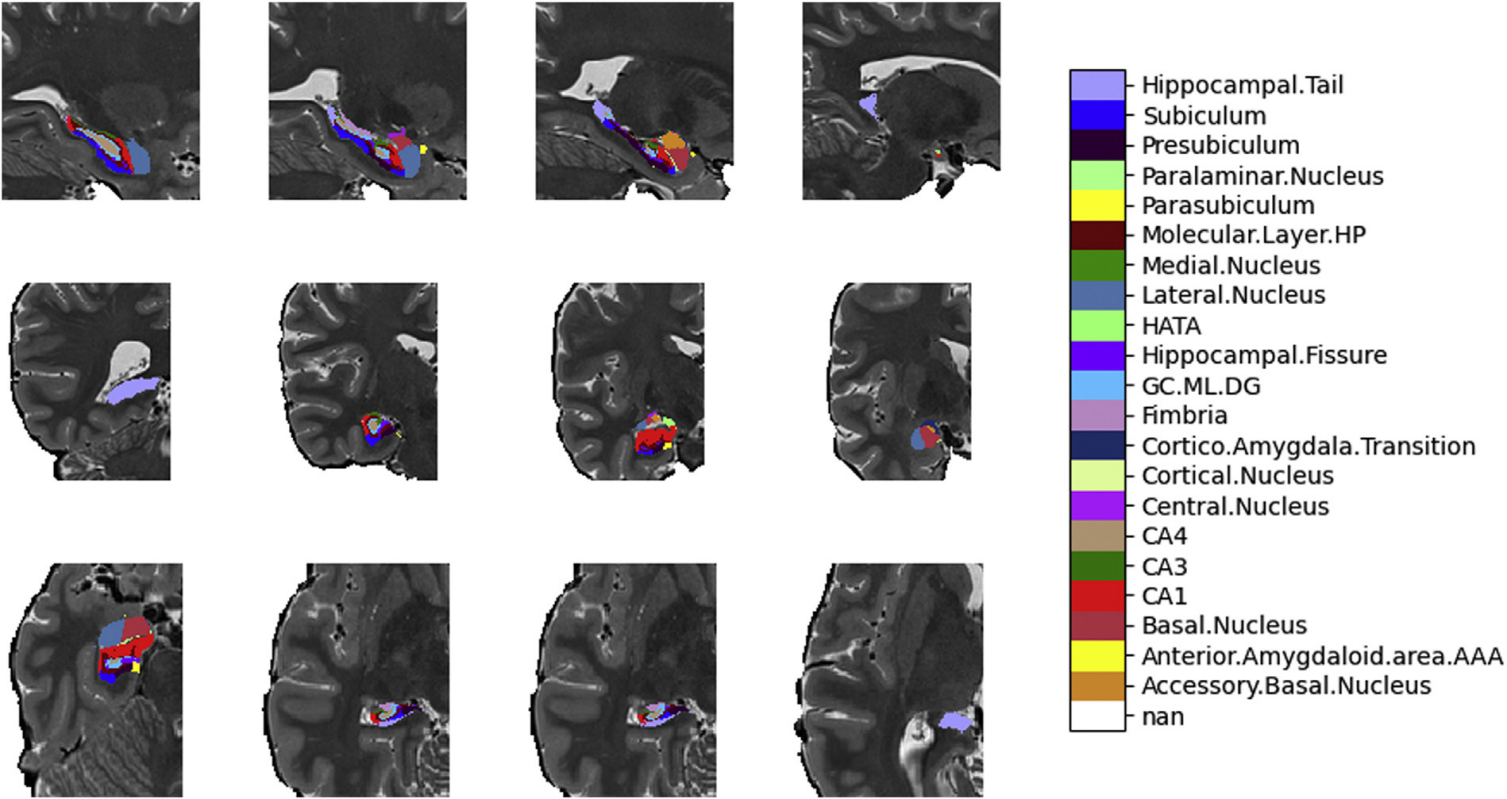
Sagittal (upper row), coronal (middle row), and axial (lower row) images of the hippocampus and the amygdala subregions performed using FreeSurfer version 6.0 subcortical reconstruction. AAA, anterior amygdaloid area; GC.ML.DG, granule and molecular cell layer of dentate gyrus; HATA, hippocampal amygdala transition area.

The reconstructions of the subcortical volumes were carried out using T1/T2-weighted images in FreeSurfer v6.0 software (http://surfer.nmr.mgh.harvard.edu/). The standard “recon-all-all” processing pipeline in FreeSurfer performed reliable automatic subcortical segmentation (Brown et al., 2020) and skull stripping after image motion correction and brain extraction on all subjects. Subsequently, the segmentation of subcortical structures was examined by a nonlinear warping atlas (31), and the volumetric estimates (32) of the following hippocampal subfields for each participant were obtained (see Fig. 1): CA1, CA2/3, CA4, presubiculum, subiculum, hippocampal tail, parasubiculum, the molecular and granule cell layers of the dentate gyrus (GC.ML.DG), the molecular layer, and the hippocampal amygdala transition area. The ENIGMA Consortium Quality Control procedure for GWAS Meta-Analysis of Subcortical Volumes (available at http://enigma.ini.usc.edu/protocols/imaging-protocols/) was followed to identify problematic boundaries in chosen subcortical regions of interest.

Preprocessed images were repurposed, and a FreeSurfer cross-sectional pipeline was used (https://surfer.nmr.mgh.harvard.edu/fswiki/HippocampalSubfieldsAndNucleiOfAmygdala) to obtain the volumetric estimates of the following amygdala subnuclei for each subject: lateral nucleus, basal nucleus, central nucleus, medial nucleus, cortical nucleus, accessory basal nucleus, corticoamygdaloid transition zone, anterior amygdaloid area, and the paralaminar nucleus. The ENIGMA QC procedure excluded 10 participants from statistical analysis due to rank violations.

### 2.3 Genetic quality control and PRS

All YA-HCP data are publicly available, including genome-wide genotype data distributed through the database of Genotypes and Phenotypes. SNPs were excluded where the minor allele frequency was less than 1% if the call rate was less than 98% or if the χ2 test for Hardy-Weinberg equilibrium had a *p* > 1 × 10^−4^. Individuals were excluded for ambiguous sex (genotypic sex and phenotypic sex not aligning) or genotyping completeness less than 97%. A total of 1,137,480 variants and 1119 individuals were considered for PRS creation. Participants that did not match the ethnicity of our discovery sample GWAS were excluded from the analysis. PRS were created using discovery data from 3 GWAS for hippocampal volume (N = 33,536; Hibar et al., 2017), amygdala volume (N = 34,431; Satizabal et al., 2019), and AD (N_CASES_ = 71,880, N_CONTROLS_ = 373,378; Jansen et al., 2020), all of which were performed on participants of Caucasian descent. For these GWAS, all SNPs with a low minor allele frequency (<0.01) were excluded. PRS were created as previously described (International Schizophrenia et al., 2009), using PRSice software version 1.25 (Euesden et al., 2015). Briefly, PRS were calculated using the “score” command in PLINK 2, which averages the number of risk alleles for each index SNP weighted by the beta coefficient for each of the 3 GWAS summary statistic files. Clumping was applied to ensure that all index SNPs were independent (500 kb, r = 0.1). SNPs within the entire APOE locus (chromosome 19: 44.4–46.5 Mb) were excluded to minimize association because of variance in APOE. Due to the complexity of the MHC region between 26 and 33 Mb on chromosome 6, SNPs in this region were also removed. For each of the 3 models, nine PRS were generated across a broad and comprehensive range of *p* value thresholds (Escott-Price et al., 2017; So and Sham, 2017). The *p* thresholds ranged from a conservative (including only GWAS significant SNPs (*p* < 1 × 10^−8^) to all independent SNPs (*p* = 1.0) at logarithmic increments (*p* < 1 × 10^−8^; *p* < 1 × 10^−7^; *p* < 1 × 10^−6^; *p* < 1 × 10^−5^; *p* < 1 × 10^−4^; *p* < 1 × 10^−3^; *p* < 1 × 10^−2^; *p* < 1 × 10^−1^). A wide *p* threshold range was chosen, as AD-PRS have been linked to case-control status and related biomarkers using a range of conservative (Ge et al., 2018; Lupton et al., 2016) and liberal *p* thresholds (Foley et al., 2017; Lancaster et al., 2019; Mormino et al., 2016). Although liberal *p* thresholds offer more predictive power in predicting case/control differences (Escott-Price et al., 2015), they contain more false positives, whereas conservative *p* thresholds are estimated with proportionally more causal SNPs (Dudbridge, 2013). The number of SNPs in each PRS model can be found in Supplementary Table 1.

### 2.4 Power analysis

Post-hoc analysis was performed using R v3.5.2 with the “pwr.r.test” function (Champely et al., 2018). The 2-sided approximate correlation power calculation with the complete imaging sample size (N =  730) at 0.05 α level and power of 80% to identify associations explaining more than 1.07% variance. Based on our prior observations between AD-PRS (excluding effects of *APOE*) and hippocampal volume in young adults and the lowest reported effect size (R^2^ = 1.6% Table 1 in a study by Foley et al., 2017), we had 93% power to detect this effect in our current sample.

### 2.5 Statistical analysis

All analyses were performed in R v3.6.1. Linear mixed-effects regression models were used to assess the influence of the PRS models on individual subfields. As related individuals were included in the sample (excluding half-siblings), a sparse kinship matrix was created to control for the familial structure. The kinship matrix was included in the LME models using the “lme4qt” package (Ziyatdinov et al., 2018), previously used to perform linear mixed models regression in samples with latent kinship structure (Hall et al., 2020; Lancaster, 2019). The AD-PRS and APOE ϵ4 status were included as fixed effects in addition to covariates, including age, sex, years of education (SSAGA_Edu), estimated total intracranial volume (eTIV: sum of the gray matter, white matter, and CSF volumes) and the top 20 principal components derived from the LD pruned data (R^2^ = 0.1; 500 kb) to correct for population stratification (Price et al., 2006). After removing participants that did not match the original GWAS sample ethnicity (N = 878), we further removed participants with (1) missing data or (2) were identified as statistical outliers using an outlier labeling rule (see below) for final sample of 730. Each MRI volumetric measure was further pruned based on an outlier detection protocol using the interquartile range outlier labeling rule (1.5 ×  interquartile range [Q3−Q1]). False discovery rate (FDR) was used to control for type I error across the whole experiment across all 414 observations, including all subregions, PRS models, and *p* thresholds.

## 3 Results

### 3.1 Hippocampus and amygdala PRS effects

**Fig. 2:**
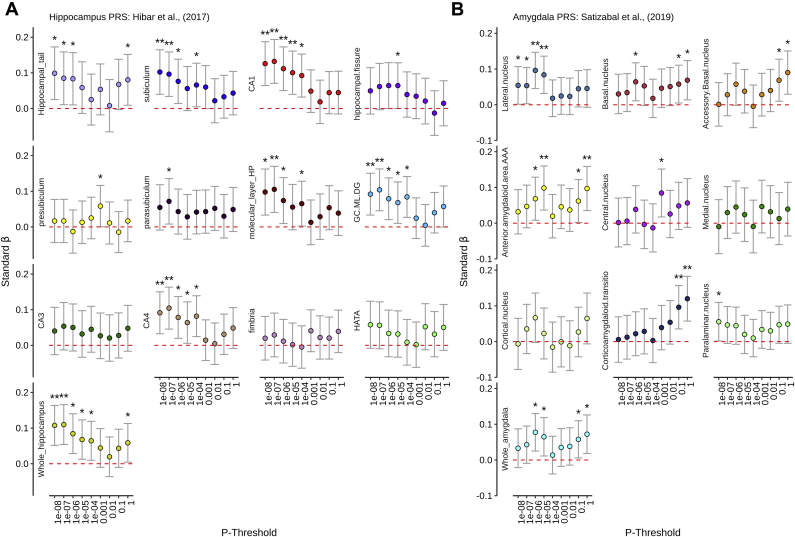
Positive control results: standardized β coefficients (Y-axis) for (A) hippocampus and (B) amygdala PRS (controlling for demographic and genetic confounds) across a range of *p* thresholds (X-axis). ∗Nominal significance (*p*_UNCORRECTED_ < 0.05), ∗∗Survival for FDR (*p*_FDR_ < 0.05).

After FDR correction across all observations, we identified several positive associations between hippocampus PRS and whole hippocampus (*p*_T_ < 1 × 10^−7^, β = 0.109, *p*_FDR_ = 0.014) and specific hippocampal subregions (including CA1, CA4, GC.ML.DG, molecular layer, subiculum). Furthermore, we identified FDR-corrected positive associations between amygdala PRS and corticoamygdaloid transition zone, anterior amygdaloid area and the lateral nucleus. Amygdala PRS was positively related to whole amygdala but was not significant after FDR correction (*p*_T_ < 1 × 10^−6^; β = 0.077, *p*_FDR_ = 0.059). See Fig. 2, Table 2, and Supplementary Table 2 for all estimates.

**Table 2:** Linear mixed model regression results for PRS on hippocampal and amygdala subfields/subnuclei volumes, corrected for genetic and demographic confounds

Region of interest	PT	BETA	SE	*p*	FDR
Hippocampus PRS (Hibar et al., 2017)					
CA1	1E-08	0.126	0.031	0.000	0.013
CA1	1E-07	0.132	0.031	0.000	0.010
CA1	1E-06	0.112	0.031	0.000	0.021
CA1	1E-05	0.100	0.031	0.001	0.045
CA4	1E-08	0.091	0.030	0.002	0.048
CA4	1E-07	0.104	0.030	0.001	0.024
GC.ML.DG	1E-08	0.092	0.030	0.002	0.047
GC.ML.DG	1E-07	0.104	0.030	0.001	0.024
Molecular layer HP	1E-07	0.105	0.033	0.001	0.045
Subiculum	1E-08	0.102	0.032	0.001	0.045
Subiculum	1E-07	0.096	0.032	0.002	0.048
Whole hippocampus	1E-08	0.108	0.029	0.000	0.014
Whole hippocampus	1E-07	0.110	0.029	0.000	0.014
Amygdala PRS (Satizabal et al., 2019)					
Anterior amygdaloid area	1E-05	0.098	0.031	0.002	0.046
Anterior amygdaloid area	1E+00	0.097	0.032	0.002	0.047
Corticoamygdaloid transition	1E-01	0.096	0.031	0.002	0.046
Corticoamygdaloid transition	1E+00	0.120	0.032	0.000	0.014
Lateral nucleus	1E-06	0.096	0.026	0.000	0.014
Lateral nucleus	1E-05	0.084	0.027	0.002	0.046
Alzheimer's PRS (Jansen et al., 2020)					
CA1	1E-04	−0.096	0.031	0.002	0.045
Hippocampal fissure	1E-05	−0.101	0.031	0.001	0.041

Key: BETA, standardized beta coefficient; FDR, *p* value corrected for false discovery rate; PRS, polygenic risk score; PT, *p* threshold; SE, standard error of beta coefficient.

### 3.2 AD PRS effects

**Fig. 3:**
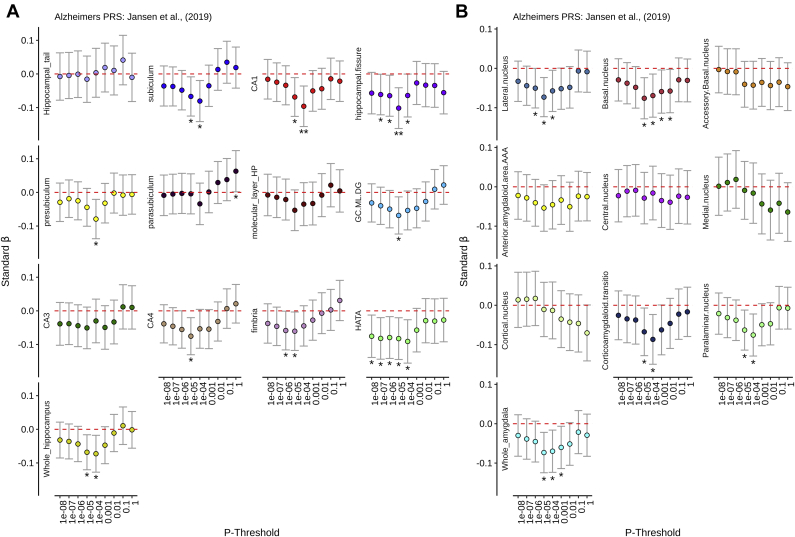
Standardized β coefficients (Y-axis) for Alzheimer's disease PRS (controlling for demographic and genetic confounds) across a range of *p* thresholds (X-axis) for (A) hippocampal and (B) amygdala subregions. ∗Nominal significance (*p*_UNCORRECTED_ < 0.05), ∗∗Survival for FDR (*p*_FDR_ < 0.05).

We first provide further evidence for a negative association between AD-PRS and whole hippocampal/amygdala volume. However, these were not significant after FDR correction (hippocampus: *p*_T_ < 1 × 10^−4^; β = −0.072, *p*_FDR_ = 0.090, amygdala: *p*_T_ < 1 × 10^−5^; β = −0.073, *p*_FDR_ = 0.069). Critically, we observe novel, FDR-corrected associations between AD-PRS and specific hippocampal subfields such as CA1 (*p*_T_ < 1 × 10^−4^; β = −0.096, *p*_FDR_ = 0.045) and the fissure (*p*_T_ < 1 × 10^−5^: β = −0.101, *p*_FDR_ = 0.041; Fig. 3). See Table 2 and Supplementary Table 3 for all estimates.

### 3.3 APOE ε4 effects

A post-hoc analysis of *APOE* ε4 absence/presence and volume of the 23 regions of interest demonstrated no associations (*p* > 0.05, in all cases). A comprehensive analysis (β, standard error of the β and *p* values, for all subregions) is documented in Supplementary Table 4.

## 4 Discussion

We aimed to ascertain the combined role of GWAS-identified SNPs on MTL subvolumes, specifically within the subfields and subnuclei of the hippocampus and amygdala, respectively. Our first objective was to provide a positive control by establishing reproducible associations between hippocampus and amygdala volumes and their respective PRS. Our observations show that hippocampus and amygdala PRS are associated with gross volume of these structures in an independent sample. Furthermore, we provide novel evidence that PRS for both the hippocampus and amygdala may be disproportionally influenced by specific regions within the hippocampal (CA1, CA4, GC.ML.DG, molecular layer , subiculum) and amygdala subregions (corticoamygdaloid transition zone, anterior amygdaloid area and the lateral nucleus). These observations show that the PRS models hold critical predictive capacity in our experimental sample, suggesting the sample had power to detect the combined effects of GWAS-identified alleles. However, we observed that different *p* thresholds yielded mixed predictive capacity, which could be explained by underpowered discovery GWAS, training discovery sample, or methodological differences.

We further observed negative associations between AD-PRS and both whole hippocampal and amygdala volumes at more conservative *p* thresholds. This is consistent with prior observations demonstrating optimal negative associations between AD-PRS (at the *p* < 0.0001 threshold) in younger (Foley et al., 2017) and older adults (Lupton et al., 2016), where the AD-PRS explained 3.9% and 0.3% in whole hippocampal volume, respectively. This *p* threshold could reflect an optimal combination of causal SNPs (less false positives) and power (from polygenic effects). The association between AD-PRS and the amygdala is also consistent with a recent GWAS of hippocampus and amygdala, showing negative genetic correlations with AD (Hibar et al., 2017; Satizabal et al., 2019) and consistent with existing evidence linking AD-PRS and MTL volume structure volume variation. More broadly, several studies have linked AD-PRS to variation in brain structure at different points across the lifespan (Axelrud et al., 2018; Foley et al., 2017; Lancaster et al., 2019; Lupton et al., 2016; Mormino et al., 2016). Our second objective was to quantify the relationship between AD-PRS and hippocampal/amygdala subfields. Hippocampal atrophy in subregions, such as CA1 and the subiculum, may be indicative markers of future AD-linked decline beyond whole hippocampal volume (Hett et al., 2019; Kerchner et al., 2010, 2012; Khan et al., 2015; La Joie et al., 2013; Vasta et al., 2016). After correcting for FDR, we observed the strongest negative associations between AD-PRS and hippocampal subregions were in CA1 and the fissure. Both CA1 and the fissure have also recently been implicated as AD-vulnerable MTL subregions (Wisse et al., 2014; Zhao et al., 2019). Atrophy within CA1 has further been linked to reduced neuronal count (Blanken et al., 2017).

The present study must be interpreted with the following considerations. First, the spatial resolution provided by the 3T MRI data. Precise subfield boundary separation is difficult at the resolution achievable within a reasonable scan time at this field strength. Although each segmentation was statistically and visually checked for segmentation errors, small volume subfields (e.g., the hippocampal amygdala transition area and parasubiculum) or thin regions (e.g., the ML.CG.DL) may be more difficult to resolve/error prone. We further note that the predictive performance of the positive control PRS (hippocampus/amygdala) was broadly superior in larger subregions. As the hippocampal/amygdala PRS are derived from whole hippocampal/amygdala volumes, sample variance may be disproportionally accounted for by the larger subregions. An alternative explanation is that the large subregions are more stable/less susceptible to artifacts in the segmentation procedure. We suggest that disproportionate measurement error across subregions could be a technical confound for all MTL segmentation studies and inference regarding smaller subregions should be interpreted with caution. Second, despite minimizing population effects by excluding non-Caucasian participants and including 20 principal components as covariates, we cannot fully exclude population stratification effects or any residual kinship structure that may influence our results. We also note that these inferences are restricted to Caucasian populations to match the discovery GWAS used as training data. We suggest that future transethnic GWAS will be necessary to broaden the generalizability of the inferences and take full advantage of ethnically mixed MRI cohorts, such as HCP. Third, it is difficult to determine the longitudinal impact of AD-PRS because of the cross-sectional design of this study. Future studies that assess development/aging will help to understand the impact of AD-PRS on MTL volumetry across the lifespan (Bookheimer et al., 2019; Harms et al., 2018; Somerville et al., 2018). However, as our sample is of young adults (aged 22–35 years), we would suggest that accumulated environmental factors linked to reduced MTL structure (e.g., smoking, diabetes [Durazzo et al., 2013; Li et al., 2020]) have limited impact, as these are accumulated over an individual's lifespan. Finally, we also note that pleotropic variants/shared genetic architecture between AD and cognition may also influence brain volumes such as the hippocampus and amygdala (Hill et al., 2016; Luciano et al., 2015; Maglanoc et al., 2020). To conclude, our observations may help to establish processes by which polygenic variation may influence specific nuclei in AD-vulnerable MTL volumes. Future studies investigating biologically informed pathways may further help us to understand how AD biomarkers are influenced by genetic risk (Ahmad et al., 2018; Lancaster et al., 2019). Furthermore, advanced multivariate GWAS may help to establish specific, shared risk loci and biological mechanisms between AD and MTL volumes (Broce et al., 2019; Yokoyama et al., 2016). Refining AD-PRS and MRI strategies for MTL subregions may help refine preclinical imaging biomarkers for AD risk detection and treatment strategies.

## Disclosure statement

The authors declare that they have no conflict of interest.

## CRediT authorship contribution statement

**Amy N. Murray:** Investigation, Visualization, Writing - review & editing. **Hannah L. Chandler:** Writing - original draft, Writing - review & editing, Supervision. **Thomas M. Lancaster:** Conceptualization, Methodology, Writing - review & editing, Supervision.
