# Supplementary material for: Multimodal hippocampal and amygdala subfield volumetry in polygenic risk for Alzheimer's disease
Source: Neurobiol Aging. 2021 Feb;98:33–41. doi: 10.1016/j.neurobiolaging.2020.08.022 (PMC7886309; doi:10.1016/j.neurobiolaging.2020.08.022)
Supplement: Supplement Table [file mmc1.docx]

**Supplementary Table 1. Number of SNPs ~ PRS**

| P-threshold | Hibar et al. 2017 | Satizabal et al., 2019 | Jansen et al., 2019 |
| --- | --- | --- | --- |
| 0.00000001 | 6 | 1 | 19 |
| 0.0000001 | 8 | 2 | 26 |
| 0.000001 | 13 | 6 | 38 |
| 0.00001 | 21 | 15 | 68 |
| 0.0001 | 84 | 66 | 182 |
| 0.001 | 565 | 508 | 731 |
| 0.01 | 3973 | 3626 | 4681 |
| 0.1 | 27273 | 25587 | 31391 |
| 1 | 141693 | 131329 | 162333 |

**Supplementary Table 2. Hippocampal / Amygdala PRS ~ Subregions**

| ROI | PTS | BETA | SE | P | FDR |
| --- | --- | --- | --- | --- | --- |
| Hippocampal_tail | 1.00E-08 | 0.099 | 0.038 | 0.009 | 0.091 |
| Hippocampal_tail | 1.00E-07 | 0.085 | 0.038 | 0.025 | 0.160 |
| Hippocampal_tail | 1.00E-06 | 0.083 | 0.037 | 0.026 | 0.162 |
| Hippocampal_tail | 1.00E-05 | 0.059 | 0.037 | 0.113 | 0.305 |
| Hippocampal_tail | 1.00E-04 | 0.025 | 0.036 | 0.493 | 0.659 |
| Hippocampal_tail | 0.001 | 0.054 | 0.036 | 0.141 | 0.346 |
| Hippocampal_tail | 0.01 | 0.008 | 0.037 | 0.834 | 0.895 |
| Hippocampal_tail | 0.1 | 0.068 | 0.036 | 0.060 | 0.241 |
| Hippocampal_tail | 1 | 0.080 | 0.036 | 0.027 | 0.167 |
| subiculum | 1.00E-08 | 0.102 | 0.032 | 0.001 | 0.045 |
| subiculum | 1.00E-07 | 0.096 | 0.032 | 0.002 | 0.048 |
| subiculum | 1.00E-06 | 0.076 | 0.032 | 0.016 | 0.124 |
| subiculum | 1.00E-05 | 0.056 | 0.031 | 0.075 | 0.265 |
| subiculum | 1.00E-04 | 0.066 | 0.031 | 0.034 | 0.188 |
| subiculum | 0.001 | 0.060 | 0.031 | 0.051 | 0.222 |
| subiculum | 0.01 | 0.022 | 0.032 | 0.490 | 0.659 |
| subiculum | 0.1 | 0.033 | 0.031 | 0.282 | 0.483 |
| subiculum | 1 | 0.043 | 0.031 | 0.164 | 0.371 |
| CA1 | 1.00E-08 | 0.126 | 0.031 | 0.000 | 0.013 |
| CA1 | 1.00E-07 | 0.132 | 0.031 | 0.000 | 0.010 |
| CA1 | 1.00E-06 | 0.112 | 0.031 | 0.000 | 0.021 |
| CA1 | 1.00E-05 | 0.100 | 0.031 | 0.001 | 0.045 |
| CA1 | 1.00E-04 | 0.092 | 0.031 | 0.003 | 0.050 |
| CA1 | 0.001 | 0.049 | 0.030 | 0.109 | 0.305 |
| CA1 | 0.01 | 0.019 | 0.031 | 0.550 | 0.702 |
| CA1 | 0.1 | 0.044 | 0.030 | 0.141 | 0.346 |
| CA1 | 1 | 0.045 | 0.031 | 0.145 | 0.350 |
| hippocampal.fissure | 1.00E-08 | 0.049 | 0.033 | 0.136 | 0.344 |
| hippocampal.fissure | 1.00E-07 | 0.062 | 0.033 | 0.063 | 0.246 |
| hippocampal.fissure | 1.00E-06 | 0.064 | 0.033 | 0.051 | 0.222 |
| hippocampal.fissure | 1.00E-05 | 0.064 | 0.033 | 0.049 | 0.222 |
| hippocampal.fissure | 1.00E-04 | 0.039 | 0.032 | 0.226 | 0.443 |
| hippocampal.fissure | 0.001 | 0.034 | 0.032 | 0.283 | 0.483 |
| hippocampal.fissure | 0.01 | 0.021 | 0.033 | 0.524 | 0.682 |
| hippocampal.fissure | 0.1 | -0.013 | 0.032 | 0.689 | 0.821 |
| hippocampal.fissure | 1 | 0.015 | 0.032 | 0.650 | 0.794 |
| presubiculum | 1.00E-08 | 0.017 | 0.031 | 0.592 | 0.749 |
| presubiculum | 1.00E-07 | 0.017 | 0.031 | 0.589 | 0.748 |
| presubiculum | 1.00E-06 | -0.013 | 0.031 | 0.672 | 0.807 |
| presubiculum | 1.00E-05 | 0.013 | 0.030 | 0.671 | 0.807 |
| presubiculum | 1.00E-04 | 0.025 | 0.030 | 0.399 | 0.582 |
| presubiculum | 0.001 | 0.058 | 0.030 | 0.049 | 0.222 |
| presubiculum | 0.01 | 0.011 | 0.030 | 0.714 | 0.830 |
| presubiculum | 0.1 | -0.015 | 0.029 | 0.620 | 0.769 |
| presubiculum | 1 | 0.017 | 0.030 | 0.573 | 0.730 |
| parasubiculum | 1.00E-08 | 0.055 | 0.032 | 0.092 | 0.280 |
| parasubiculum | 1.00E-07 | 0.072 | 0.032 | 0.027 | 0.167 |
| parasubiculum | 1.00E-06 | 0.043 | 0.032 | 0.182 | 0.391 |
| parasubiculum | 1.00E-05 | 0.028 | 0.032 | 0.378 | 0.565 |
| parasubiculum | 1.00E-04 | 0.042 | 0.031 | 0.183 | 0.391 |
| parasubiculum | 0.001 | 0.043 | 0.031 | 0.165 | 0.371 |
| parasubiculum | 0.01 | 0.052 | 0.032 | 0.099 | 0.292 |
| parasubiculum | 0.1 | 0.030 | 0.031 | 0.336 | 0.535 |
| parasubiculum | 1 | 0.049 | 0.032 | 0.121 | 0.320 |
| molecular_layer_HP | 1.00E-08 | 0.098 | 0.033 | 0.003 | 0.053 |
| molecular_layer_HP | 1.00E-07 | 0.105 | 0.033 | 0.001 | 0.045 |
| molecular_layer_HP | 1.00E-06 | 0.074 | 0.033 | 0.025 | 0.160 |
| molecular_layer_HP | 1.00E-05 | 0.056 | 0.033 | 0.087 | 0.276 |
| molecular_layer_HP | 1.00E-04 | 0.065 | 0.032 | 0.043 | 0.210 |
| molecular_layer_HP | 0.001 | 0.013 | 0.032 | 0.690 | 0.821 |
| molecular_layer_HP | 0.01 | 0.029 | 0.033 | 0.377 | 0.565 |
| molecular_layer_HP | 0.1 | 0.054 | 0.032 | 0.087 | 0.276 |
| molecular_layer_HP | 1 | 0.039 | 0.032 | 0.226 | 0.443 |
| GC.ML.DG | 1.00E-08 | 0.092 | 0.030 | 0.002 | 0.047 |
| GC.ML.DG | 1.00E-07 | 0.104 | 0.030 | 0.001 | 0.024 |
| GC.ML.DG | 1.00E-06 | 0.079 | 0.030 | 0.008 | 0.091 |
| GC.ML.DG | 1.00E-05 | 0.068 | 0.030 | 0.022 | 0.149 |
| GC.ML.DG | 1.00E-04 | 0.084 | 0.029 | 0.004 | 0.059 |
| GC.ML.DG | 0.001 | 0.025 | 0.029 | 0.399 | 0.582 |
| GC.ML.DG | 0.01 | 0.004 | 0.030 | 0.883 | 0.928 |
| GC.ML.DG | 0.1 | 0.040 | 0.029 | 0.170 | 0.377 |
| GC.ML.DG | 1 | 0.057 | 0.029 | 0.052 | 0.222 |
| CA3 | 1.00E-08 | 0.040 | 0.034 | 0.236 | 0.451 |
| CA3 | 1.00E-07 | 0.054 | 0.034 | 0.115 | 0.305 |
| CA3 | 1.00E-06 | 0.050 | 0.034 | 0.137 | 0.344 |
| CA3 | 1.00E-05 | 0.032 | 0.033 | 0.341 | 0.536 |
| CA3 | 1.00E-04 | 0.045 | 0.033 | 0.170 | 0.377 |
| CA3 | 0.001 | 0.027 | 0.033 | 0.415 | 0.596 |
| CA3 | 0.01 | 0.020 | 0.033 | 0.538 | 0.691 |
| CA3 | 0.1 | 0.028 | 0.032 | 0.390 | 0.574 |
| CA3 | 1 | 0.048 | 0.033 | 0.145 | 0.350 |
| CA4 | 1.00E-08 | 0.091 | 0.030 | 0.002 | 0.048 |
| CA4 | 1.00E-07 | 0.104 | 0.030 | 0.001 | 0.024 |
| CA4 | 1.00E-06 | 0.078 | 0.030 | 0.009 | 0.091 |
| CA4 | 1.00E-05 | 0.064 | 0.030 | 0.032 | 0.181 |
| CA4 | 1.00E-04 | 0.082 | 0.029 | 0.005 | 0.070 |
| CA4 | 0.001 | 0.014 | 0.029 | 0.620 | 0.769 |
| CA4 | 0.01 | 0.005 | 0.030 | 0.879 | 0.926 |
| CA4 | 0.1 | 0.031 | 0.029 | 0.283 | 0.483 |
| CA4 | 1 | 0.048 | 0.029 | 0.097 | 0.290 |
| fimbria | 1.00E-08 | 0.020 | 0.031 | 0.524 | 0.682 |
| fimbria | 1.00E-07 | 0.029 | 0.031 | 0.354 | 0.547 |
| fimbria | 1.00E-06 | 0.012 | 0.031 | 0.710 | 0.830 |
| fimbria | 1.00E-05 | 0.002 | 0.031 | 0.955 | 0.968 |
| fimbria | 1.00E-04 | -0.005 | 0.030 | 0.874 | 0.923 |
| fimbria | 0.001 | 0.041 | 0.030 | 0.173 | 0.378 |
| fimbria | 0.01 | 0.022 | 0.031 | 0.478 | 0.655 |
| fimbria | 0.1 | 0.020 | 0.030 | 0.498 | 0.663 |
| fimbria | 1 | 0.039 | 0.030 | 0.199 | 0.418 |
| HATA | 1.00E-08 | 0.058 | 0.034 | 0.086 | 0.276 |
| HATA | 1.00E-07 | 0.056 | 0.034 | 0.094 | 0.283 |
| HATA | 1.00E-06 | 0.033 | 0.033 | 0.317 | 0.511 |
| HATA | 1.00E-05 | 0.032 | 0.033 | 0.339 | 0.536 |
| HATA | 1.00E-04 | 0.008 | 0.033 | 0.795 | 0.878 |
| HATA | 0.001 | 0.002 | 0.032 | 0.955 | 0.968 |
| HATA | 0.01 | 0.052 | 0.033 | 0.115 | 0.305 |
| HATA | 0.1 | 0.031 | 0.032 | 0.335 | 0.535 |
| HATA | 1 | 0.050 | 0.033 | 0.125 | 0.324 |
| Whole_hippocampus | 1.00E-08 | 0.108 | 0.029 | 0.000 | 0.014 |
| Whole_hippocampus | 1.00E-07 | 0.110 | 0.029 | 0.000 | 0.014 |
| Whole_hippocampus | 1.00E-06 | 0.084 | 0.028 | 0.003 | 0.053 |
| Whole_hippocampus | 1.00E-05 | 0.068 | 0.028 | 0.016 | 0.124 |
| Whole_hippocampus | 1.00E-04 | 0.064 | 0.028 | 0.021 | 0.147 |
| Whole_hippocampus | 0.001 | 0.044 | 0.028 | 0.110 | 0.305 |
| Whole_hippocampus | 0.01 | 0.019 | 0.028 | 0.493 | 0.659 |
| Whole_hippocampus | 0.1 | 0.043 | 0.027 | 0.114 | 0.305 |
| Whole_hippocampus | 1 | 0.059 | 0.028 | 0.034 | 0.188 |
| Lateral.nucleus | 1.00E-08 | 0.054 | 0.027 | 0.044 | 0.210 |
| Lateral.nucleus | 1.00E-07 | 0.054 | 0.026 | 0.038 | 0.197 |
| Lateral.nucleus | 1.00E-06 | 0.096 | 0.026 | 0.000 | 0.014 |
| Lateral.nucleus | 1.00E-05 | 0.084 | 0.027 | 0.002 | 0.046 |
| Lateral.nucleus | 1.00E-04 | 0.018 | 0.026 | 0.486 | 0.659 |
| Lateral.nucleus | 0.001 | 0.025 | 0.026 | 0.347 | 0.544 |
| Lateral.nucleus | 0.01 | 0.024 | 0.026 | 0.365 | 0.553 |
| Lateral.nucleus | 0.1 | 0.045 | 0.026 | 0.084 | 0.276 |
| Lateral.nucleus | 1 | 0.046 | 0.027 | 0.089 | 0.280 |
| Basal.nucleus | 1.00E-08 | 0.030 | 0.028 | 0.282 | 0.483 |
| Basal.nucleus | 1.00E-07 | 0.034 | 0.027 | 0.208 | 0.430 |
| Basal.nucleus | 1.00E-06 | 0.064 | 0.027 | 0.018 | 0.133 |
| Basal.nucleus | 1.00E-05 | 0.053 | 0.028 | 0.059 | 0.239 |
| Basal.nucleus | 1.00E-04 | 0.018 | 0.027 | 0.511 | 0.672 |
| Basal.nucleus | 0.001 | 0.046 | 0.027 | 0.092 | 0.280 |
| Basal.nucleus | 0.01 | 0.051 | 0.027 | 0.061 | 0.241 |
| Basal.nucleus | 0.1 | 0.058 | 0.027 | 0.032 | 0.181 |
| Basal.nucleus | 1 | 0.069 | 0.028 | 0.014 | 0.115 |
| Accessory.Basal.nucleus | 1.00E-08 | 0.001 | 0.031 | 0.971 | 0.975 |
| Accessory.Basal.nucleus | 1.00E-07 | 0.028 | 0.030 | 0.349 | 0.545 |
| Accessory.Basal.nucleus | 1.00E-06 | 0.057 | 0.030 | 0.056 | 0.234 |
| Accessory.Basal.nucleus | 1.00E-05 | 0.038 | 0.031 | 0.223 | 0.443 |
| Accessory.Basal.nucleus | 1.00E-04 | -0.004 | 0.030 | 0.885 | 0.928 |
| Accessory.Basal.nucleus | 0.001 | 0.028 | 0.030 | 0.352 | 0.545 |
| Accessory.Basal.nucleus | 0.01 | 0.040 | 0.030 | 0.185 | 0.392 |
| Accessory.Basal.nucleus | 0.1 | 0.069 | 0.030 | 0.022 | 0.150 |
| Accessory.Basal.nucleus | 1 | 0.090 | 0.031 | 0.004 | 0.059 |
| Anterior.amygdaloid.area.AAA | 1.00E-08 | 0.032 | 0.032 | 0.313 | 0.510 |
| Anterior.amygdaloid.area.AAA | 1.00E-07 | 0.047 | 0.030 | 0.125 | 0.324 |
| Anterior.amygdaloid.area.AAA | 1.00E-06 | 0.068 | 0.031 | 0.026 | 0.162 |
| Anterior.amygdaloid.area.AAA | 1.00E-05 | 0.098 | 0.031 | 0.002 | 0.046 |
| Anterior.amygdaloid.area.AAA | 1.00E-04 | 0.020 | 0.031 | 0.528 | 0.685 |
| Anterior.amygdaloid.area.AAA | 0.001 | 0.046 | 0.031 | 0.141 | 0.346 |
| Anterior.amygdaloid.area.AAA | 0.01 | 0.037 | 0.031 | 0.226 | 0.443 |
| Anterior.amygdaloid.area.AAA | 0.1 | 0.062 | 0.031 | 0.043 | 0.210 |
| Anterior.amygdaloid.area.AAA | 1 | 0.097 | 0.032 | 0.002 | 0.047 |
| Central.nucleus | 1.00E-08 | 0.002 | 0.035 | 0.957 | 0.968 |
| Central.nucleus | 1.00E-07 | 0.006 | 0.034 | 0.862 | 0.915 |
| Central.nucleus | 1.00E-06 | 0.039 | 0.034 | 0.253 | 0.472 |
| Central.nucleus | 1.00E-05 | -0.003 | 0.035 | 0.924 | 0.950 |
| Central.nucleus | 1.00E-04 | -0.013 | 0.034 | 0.703 | 0.826 |
| Central.nucleus | 0.001 | 0.084 | 0.034 | 0.013 | 0.113 |
| Central.nucleus | 0.01 | 0.026 | 0.034 | 0.447 | 0.627 |
| Central.nucleus | 0.1 | 0.048 | 0.034 | 0.152 | 0.358 |
| Central.nucleus | 1 | 0.057 | 0.035 | 0.103 | 0.299 |
| Medial.nucleus | 1.00E-08 | -0.010 | 0.039 | 0.801 | 0.879 |
| Medial.nucleus | 1.00E-07 | 0.030 | 0.037 | 0.421 | 0.601 |
| Medial.nucleus | 1.00E-06 | 0.045 | 0.037 | 0.226 | 0.443 |
| Medial.nucleus | 1.00E-05 | 0.024 | 0.038 | 0.532 | 0.687 |
| Medial.nucleus | 1.00E-04 | -0.009 | 0.038 | 0.805 | 0.879 |
| Medial.nucleus | 0.001 | 0.047 | 0.038 | 0.217 | 0.437 |
| Medial.nucleus | 0.01 | 0.032 | 0.037 | 0.391 | 0.574 |
| Medial.nucleus | 0.1 | 0.013 | 0.037 | 0.730 | 0.842 |
| Medial.nucleus | 1 | 0.039 | 0.038 | 0.305 | 0.507 |
| Cortical.nucleus | 1.00E-08 | -0.006 | 0.037 | 0.862 | 0.915 |
| Cortical.nucleus | 1.00E-07 | 0.035 | 0.035 | 0.314 | 0.510 |
| Cortical.nucleus | 1.00E-06 | 0.067 | 0.035 | 0.059 | 0.239 |
| Cortical.nucleus | 1.00E-05 | 0.023 | 0.036 | 0.533 | 0.687 |
| Cortical.nucleus | 1.00E-04 | -0.016 | 0.036 | 0.664 | 0.804 |
| Cortical.nucleus | 0.001 | -0.001 | 0.036 | 0.986 | 0.986 |
| Cortical.nucleus | 0.01 | -0.011 | 0.036 | 0.749 | 0.858 |
| Cortical.nucleus | 0.1 | 0.027 | 0.035 | 0.448 | 0.627 |
| Cortical.nucleus | 1 | 0.065 | 0.036 | 0.074 | 0.265 |
| Corticoamygdaloid.transitio | 1.00E-08 | 0.006 | 0.032 | 0.860 | 0.915 |
| Corticoamygdaloid.transitio | 1.00E-07 | 0.012 | 0.031 | 0.694 | 0.823 |
| Corticoamygdaloid.transitio | 1.00E-06 | 0.022 | 0.031 | 0.484 | 0.659 |
| Corticoamygdaloid.transitio | 1.00E-05 | 0.029 | 0.032 | 0.375 | 0.565 |
| Corticoamygdaloid.transitio | 1.00E-04 | 0.003 | 0.032 | 0.926 | 0.950 |
| Corticoamygdaloid.transitio | 0.001 | 0.039 | 0.032 | 0.216 | 0.437 |
| Corticoamygdaloid.transitio | 0.01 | 0.054 | 0.031 | 0.083 | 0.276 |
| Corticoamygdaloid.transitio | 0.1 | 0.096 | 0.031 | 0.002 | 0.046 |
| Corticoamygdaloid.transitio | 1 | 0.120 | 0.032 | 0.000 | 0.014 |
| Paralaminar.nucleus | 1.00E-08 | 0.056 | 0.028 | 0.043 | 0.210 |
| Paralaminar.nucleus | 1.00E-07 | 0.046 | 0.026 | 0.077 | 0.269 |
| Paralaminar.nucleus | 1.00E-06 | 0.044 | 0.027 | 0.094 | 0.283 |
| Paralaminar.nucleus | 1.00E-05 | 0.020 | 0.027 | 0.462 | 0.642 |
| Paralaminar.nucleus | 1.00E-04 | 0.010 | 0.027 | 0.713 | 0.830 |
| Paralaminar.nucleus | 0.001 | 0.034 | 0.027 | 0.206 | 0.430 |
| Paralaminar.nucleus | 0.01 | 0.030 | 0.027 | 0.258 | 0.476 |
| Paralaminar.nucleus | 0.1 | 0.047 | 0.026 | 0.075 | 0.265 |
| Paralaminar.nucleus | 1 | 0.049 | 0.027 | 0.074 | 0.265 |
| Whole_amygdala | 1.00E-08 | 0.033 | 0.028 | 0.235 | 0.451 |
| Whole_amygdala | 1.00E-07 | 0.043 | 0.027 | 0.107 | 0.304 |
| Whole_amygdala | 1.00E-06 | 0.077 | 0.027 | 0.004 | 0.059 |
| Whole_amygdala | 1.00E-05 | 0.065 | 0.027 | 0.018 | 0.133 |
| Whole_amygdala | 1.00E-04 | 0.014 | 0.027 | 0.611 | 0.764 |
| Whole_amygdala | 0.001 | 0.035 | 0.027 | 0.192 | 0.406 |
| Whole_amygdala | 0.01 | 0.038 | 0.027 | 0.155 | 0.364 |
| Whole_amygdala | 0.1 | 0.058 | 0.027 | 0.030 | 0.172 |
| Whole_amygdala | 1 | 0.072 | 0.027 | 0.009 | 0.091 |

**Supplementary Table 3. Alzhiemer’s PRS ~ Subregions**

| ROI | PTS | BETA | SE | P | FDR |
| --- | --- | --- | --- | --- | --- |
| Hippocampal_tail | 1.00E-08 | -0.008 | 0.036 | 0.831 | 0.893 |
| Hippocampal_tail | 1.00E-07 | -0.005 | 0.035 | 0.896 | 0.932 |
| Hippocampal_tail | 1.00E-06 | -0.001 | 0.035 | 0.982 | 0.984 |
| Hippocampal_tail | 1.00E-05 | -0.016 | 0.035 | 0.656 | 0.799 |
| Hippocampal_tail | 1.00E-04 | 0.003 | 0.037 | 0.926 | 0.950 |
| Hippocampal_tail | 0.001 | 0.019 | 0.037 | 0.609 | 0.764 |
| Hippocampal_tail | 0.01 | 0.010 | 0.037 | 0.779 | 0.874 |
| Hippocampal_tail | 0.1 | 0.041 | 0.038 | 0.273 | 0.483 |
| Hippocampal_tail | 1 | -0.010 | 0.037 | 0.781 | 0.874 |
| subiculum | 1.00E-08 | -0.036 | 0.030 | 0.239 | 0.454 |
| subiculum | 1.00E-07 | -0.037 | 0.030 | 0.210 | 0.430 |
| subiculum | 1.00E-06 | -0.048 | 0.030 | 0.107 | 0.304 |
| subiculum | 1.00E-05 | -0.067 | 0.030 | 0.024 | 0.160 |
| subiculum | 1.00E-04 | -0.081 | 0.031 | 0.010 | 0.092 |
| subiculum | 0.001 | -0.035 | 0.031 | 0.261 | 0.481 |
| subiculum | 0.01 | 0.013 | 0.032 | 0.673 | 0.807 |
| subiculum | 0.1 | 0.035 | 0.032 | 0.274 | 0.483 |
| subiculum | 1 | 0.019 | 0.031 | 0.544 | 0.698 |
| CA1 | 1.00E-08 | -0.016 | 0.030 | 0.600 | 0.756 |
| CA1 | 1.00E-07 | -0.025 | 0.029 | 0.402 | 0.585 |
| CA1 | 1.00E-06 | -0.034 | 0.030 | 0.254 | 0.472 |
| CA1 | 1.00E-05 | -0.069 | 0.029 | 0.020 | 0.141 |
| CA1 | 1.00E-04 | -0.096 | 0.031 | 0.002 | 0.046 |
| CA1 | 0.001 | -0.050 | 0.031 | 0.103 | 0.299 |
| CA1 | 0.01 | -0.044 | 0.031 | 0.156 | 0.364 |
| CA1 | 0.1 | -0.015 | 0.031 | 0.643 | 0.790 |
| CA1 | 1 | -0.022 | 0.031 | 0.483 | 0.659 |
| hippocampal.fissure | 1.00E-08 | -0.057 | 0.031 | 0.070 | 0.262 |
| hippocampal.fissure | 1.00E-07 | -0.061 | 0.031 | 0.047 | 0.221 |
| hippocampal.fissure | 1.00E-06 | -0.065 | 0.031 | 0.037 | 0.197 |
| hippocampal.fissure | 1.00E-05 | -0.102 | 0.031 | 0.001 | 0.042 |
| hippocampal.fissure | 1.00E-04 | -0.064 | 0.033 | 0.049 | 0.222 |
| hippocampal.fissure | 0.001 | -0.027 | 0.033 | 0.413 | 0.596 |
| hippocampal.fissure | 0.01 | -0.033 | 0.033 | 0.309 | 0.510 |
| hippocampal.fissure | 0.1 | -0.034 | 0.033 | 0.299 | 0.500 |
| hippocampal.fissure | 1 | -0.056 | 0.032 | 0.085 | 0.276 |
| presubiculum | 1.00E-08 | -0.029 | 0.029 | 0.314 | 0.510 |
| presubiculum | 1.00E-07 | -0.019 | 0.028 | 0.509 | 0.672 |
| presubiculum | 1.00E-06 | -0.025 | 0.029 | 0.385 | 0.571 |
| presubiculum | 1.00E-05 | -0.044 | 0.029 | 0.122 | 0.320 |
| presubiculum | 1.00E-04 | -0.079 | 0.030 | 0.008 | 0.091 |
| presubiculum | 0.001 | -0.032 | 0.030 | 0.285 | 0.483 |
| presubiculum | 0.01 | -0.002 | 0.030 | 0.947 | 0.968 |
| presubiculum | 0.1 | -0.008 | 0.031 | 0.791 | 0.876 |
| presubiculum | 1 | -0.006 | 0.030 | 0.839 | 0.898 |
| parasubiculum | 1.00E-08 | -0.009 | 0.031 | 0.777 | 0.874 |
| parasubiculum | 1.00E-07 | -0.005 | 0.030 | 0.871 | 0.922 |
| parasubiculum | 1.00E-06 | -0.003 | 0.030 | 0.922 | 0.950 |
| parasubiculum | 1.00E-05 | -0.004 | 0.030 | 0.887 | 0.928 |
| parasubiculum | 1.00E-04 | -0.034 | 0.032 | 0.279 | 0.483 |
| parasubiculum | 0.001 | 0.001 | 0.032 | 0.969 | 0.975 |
| parasubiculum | 0.01 | 0.029 | 0.032 | 0.358 | 0.551 |
| parasubiculum | 0.1 | 0.038 | 0.032 | 0.235 | 0.451 |
| parasubiculum | 1 | 0.063 | 0.031 | 0.044 | 0.210 |
| molecular_layer_HP | 1.00E-08 | -0.008 | 0.032 | 0.807 | 0.879 |
| molecular_layer_HP | 1.00E-07 | -0.015 | 0.031 | 0.638 | 0.786 |
| molecular_layer_HP | 1.00E-06 | -0.021 | 0.031 | 0.494 | 0.659 |
| molecular_layer_HP | 1.00E-05 | -0.053 | 0.031 | 0.086 | 0.276 |
| molecular_layer_HP | 1.00E-04 | -0.035 | 0.033 | 0.283 | 0.483 |
| molecular_layer_HP | 0.001 | -0.033 | 0.033 | 0.310 | 0.510 |
| molecular_layer_HP | 0.01 | -0.008 | 0.033 | 0.803 | 0.879 |
| molecular_layer_HP | 0.1 | 0.021 | 0.033 | 0.514 | 0.674 |
| molecular_layer_HP | 1 | 0.004 | 0.032 | 0.891 | 0.929 |
| GC.ML.DG | 1.00E-08 | -0.031 | 0.029 | 0.280 | 0.483 |
| GC.ML.DG | 1.00E-07 | -0.040 | 0.028 | 0.159 | 0.365 |
| GC.ML.DG | 1.00E-06 | -0.050 | 0.028 | 0.080 | 0.275 |
| GC.ML.DG | 1.00E-05 | -0.069 | 0.028 | 0.015 | 0.120 |
| GC.ML.DG | 1.00E-04 | -0.054 | 0.029 | 0.069 | 0.262 |
| GC.ML.DG | 0.001 | -0.047 | 0.030 | 0.110 | 0.305 |
| GC.ML.DG | 0.01 | -0.027 | 0.030 | 0.364 | 0.553 |
| GC.ML.DG | 0.1 | 0.009 | 0.030 | 0.755 | 0.859 |
| GC.ML.DG | 1 | 0.022 | 0.029 | 0.453 | 0.632 |
| CA3 | 1.00E-08 | -0.039 | 0.032 | 0.234 | 0.451 |
| CA3 | 1.00E-07 | -0.038 | 0.032 | 0.227 | 0.443 |
| CA3 | 1.00E-06 | -0.044 | 0.032 | 0.164 | 0.371 |
| CA3 | 1.00E-05 | -0.051 | 0.032 | 0.108 | 0.305 |
| CA3 | 1.00E-04 | -0.030 | 0.033 | 0.365 | 0.553 |
| CA3 | 0.001 | -0.049 | 0.033 | 0.135 | 0.343 |
| CA3 | 0.01 | -0.033 | 0.033 | 0.319 | 0.512 |
| CA3 | 0.1 | 0.012 | 0.034 | 0.724 | 0.838 |
| CA3 | 1 | 0.010 | 0.033 | 0.755 | 0.859 |
| CA4 | 1.00E-08 | -0.039 | 0.029 | 0.183 | 0.391 |
| CA4 | 1.00E-07 | -0.046 | 0.028 | 0.104 | 0.300 |
| CA4 | 1.00E-06 | -0.055 | 0.028 | 0.051 | 0.222 |
| CA4 | 1.00E-05 | -0.075 | 0.028 | 0.007 | 0.087 |
| CA4 | 1.00E-04 | -0.053 | 0.029 | 0.070 | 0.262 |
| CA4 | 0.001 | -0.054 | 0.030 | 0.066 | 0.255 |
| CA4 | 0.01 | -0.032 | 0.030 | 0.287 | 0.483 |
| CA4 | 0.1 | 0.007 | 0.030 | 0.822 | 0.887 |
| CA4 | 1 | 0.021 | 0.029 | 0.471 | 0.650 |
| fimbria | 1.00E-08 | -0.038 | 0.030 | 0.209 | 0.430 |
| fimbria | 1.00E-07 | -0.046 | 0.029 | 0.114 | 0.305 |
| fimbria | 1.00E-06 | -0.059 | 0.029 | 0.046 | 0.220 |
| fimbria | 1.00E-05 | -0.061 | 0.029 | 0.038 | 0.197 |
| fimbria | 1.00E-04 | -0.045 | 0.031 | 0.143 | 0.349 |
| fimbria | 0.001 | -0.028 | 0.030 | 0.365 | 0.553 |
| fimbria | 0.01 | -0.007 | 0.031 | 0.823 | 0.887 |
| fimbria | 0.1 | 0.003 | 0.031 | 0.927 | 0.950 |
| fimbria | 1 | 0.031 | 0.031 | 0.312 | 0.510 |
| HATA | 1.00E-08 | -0.076 | 0.032 | 0.019 | 0.137 |
| HATA | 1.00E-07 | -0.082 | 0.032 | 0.009 | 0.091 |
| HATA | 1.00E-06 | -0.079 | 0.032 | 0.012 | 0.108 |
| HATA | 1.00E-05 | -0.083 | 0.032 | 0.009 | 0.091 |
| HATA | 1.00E-04 | -0.091 | 0.033 | 0.006 | 0.072 |
| HATA | 0.001 | -0.057 | 0.033 | 0.083 | 0.276 |
| HATA | 0.01 | -0.029 | 0.034 | 0.384 | 0.571 |
| HATA | 0.1 | -0.030 | 0.034 | 0.367 | 0.555 |
| HATA | 1 | -0.027 | 0.033 | 0.408 | 0.590 |
| Whole_hippocampus | 1.00E-08 | -0.032 | 0.027 | 0.240 | 0.454 |
| Whole_hippocampus | 1.00E-07 | -0.036 | 0.026 | 0.174 | 0.378 |
| Whole_hippocampus | 1.00E-06 | -0.044 | 0.027 | 0.103 | 0.299 |
| Whole_hippocampus | 1.00E-05 | -0.068 | 0.027 | 0.010 | 0.094 |
| Whole_hippocampus | 1.00E-04 | -0.073 | 0.028 | 0.009 | 0.091 |
| Whole_hippocampus | 0.001 | -0.047 | 0.028 | 0.092 | 0.280 |
| Whole_hippocampus | 0.01 | -0.011 | 0.028 | 0.696 | 0.824 |
| Whole_hippocampus | 0.1 | 0.011 | 0.028 | 0.710 | 0.830 |
| Whole_hippocampus | 1 | -0.002 | 0.028 | 0.954 | 0.968 |
| Lateral.nucleus | 1.00E-08 | -0.033 | 0.026 | 0.209 | 0.430 |
| Lateral.nucleus | 1.00E-07 | -0.044 | 0.025 | 0.082 | 0.276 |
| Lateral.nucleus | 1.00E-06 | -0.050 | 0.026 | 0.050 | 0.222 |
| Lateral.nucleus | 1.00E-05 | -0.073 | 0.025 | 0.004 | 0.059 |
| Lateral.nucleus | 1.00E-04 | -0.057 | 0.027 | 0.032 | 0.181 |
| Lateral.nucleus | 0.001 | -0.052 | 0.027 | 0.053 | 0.222 |
| Lateral.nucleus | 0.01 | -0.048 | 0.027 | 0.073 | 0.265 |
| Lateral.nucleus | 0.1 | -0.007 | 0.027 | 0.806 | 0.879 |
| Lateral.nucleus | 1 | -0.008 | 0.027 | 0.750 | 0.858 |
| Basal.nucleus | 1.00E-08 | -0.029 | 0.027 | 0.285 | 0.483 |
| Basal.nucleus | 1.00E-07 | -0.038 | 0.027 | 0.157 | 0.364 |
| Basal.nucleus | 1.00E-06 | -0.048 | 0.027 | 0.073 | 0.265 |
| Basal.nucleus | 1.00E-05 | -0.076 | 0.027 | 0.004 | 0.062 |
| Basal.nucleus | 1.00E-04 | -0.069 | 0.028 | 0.013 | 0.113 |
| Basal.nucleus | 0.001 | -0.059 | 0.028 | 0.035 | 0.189 |
| Basal.nucleus | 0.01 | -0.058 | 0.028 | 0.040 | 0.204 |
| Basal.nucleus | 0.1 | -0.029 | 0.028 | 0.304 | 0.507 |
| Basal.nucleus | 1 | -0.031 | 0.028 | 0.270 | 0.483 |
| Accessory.Basal.nucleus | 1.00E-08 | -0.003 | 0.030 | 0.920 | 0.950 |
| Accessory.Basal.nucleus | 1.00E-07 | -0.008 | 0.030 | 0.785 | 0.875 |
| Accessory.Basal.nucleus | 1.00E-06 | -0.009 | 0.030 | 0.770 | 0.869 |
| Accessory.Basal.nucleus | 1.00E-05 | -0.040 | 0.030 | 0.173 | 0.378 |
| Accessory.Basal.nucleus | 1.00E-04 | -0.043 | 0.031 | 0.168 | 0.375 |
| Accessory.Basal.nucleus | 0.001 | -0.036 | 0.031 | 0.250 | 0.468 |
| Accessory.Basal.nucleus | 0.01 | -0.044 | 0.031 | 0.162 | 0.371 |
| Accessory.Basal.nucleus | 0.1 | -0.035 | 0.032 | 0.268 | 0.483 |
| Accessory.Basal.nucleus | 1 | -0.046 | 0.031 | 0.133 | 0.341 |
| Anterior.amygdaloid.area.AAA | 1.00E-08 | -0.022 | 0.031 | 0.473 | 0.651 |
| Anterior.amygdaloid.area.AAA | 1.00E-07 | -0.028 | 0.030 | 0.350 | 0.545 |
| Anterior.amygdaloid.area.AAA | 1.00E-06 | -0.041 | 0.030 | 0.181 | 0.391 |
| Anterior.amygdaloid.area.AAA | 1.00E-05 | -0.054 | 0.030 | 0.074 | 0.265 |
| Anterior.amygdaloid.area.AAA | 1.00E-04 | -0.046 | 0.031 | 0.146 | 0.350 |
| Anterior.amygdaloid.area.AAA | 0.001 | -0.034 | 0.032 | 0.287 | 0.483 |
| Anterior.amygdaloid.area.AAA | 0.01 | -0.051 | 0.032 | 0.112 | 0.305 |
| Anterior.amygdaloid.area.AAA | 0.1 | -0.024 | 0.032 | 0.463 | 0.642 |
| Anterior.amygdaloid.area.AAA | 1 | -0.025 | 0.031 | 0.424 | 0.603 |
| Central.nucleus | 1.00E-08 | -0.023 | 0.034 | 0.505 | 0.668 |
| Central.nucleus | 1.00E-07 | -0.011 | 0.033 | 0.739 | 0.849 |
| Central.nucleus | 1.00E-06 | -0.009 | 0.033 | 0.788 | 0.875 |
| Central.nucleus | 1.00E-05 | -0.029 | 0.033 | 0.390 | 0.574 |
| Central.nucleus | 1.00E-04 | -0.016 | 0.035 | 0.650 | 0.794 |
| Central.nucleus | 0.001 | -0.035 | 0.035 | 0.316 | 0.511 |
| Central.nucleus | 0.01 | -0.039 | 0.035 | 0.265 | 0.481 |
| Central.nucleus | 0.1 | -0.024 | 0.035 | 0.501 | 0.665 |
| Central.nucleus | 1 | -0.027 | 0.035 | 0.442 | 0.624 |
| Medial.nucleus | 1.00E-08 | 0.001 | 0.038 | 0.969 | 0.975 |
| Medial.nucleus | 1.00E-07 | 0.011 | 0.037 | 0.767 | 0.868 |
| Medial.nucleus | 1.00E-06 | 0.019 | 0.037 | 0.616 | 0.768 |
| Medial.nucleus | 1.00E-05 | -0.009 | 0.038 | 0.809 | 0.880 |
| Medial.nucleus | 1.00E-04 | -0.016 | 0.039 | 0.682 | 0.816 |
| Medial.nucleus | 0.001 | -0.043 | 0.039 | 0.265 | 0.481 |
| Medial.nucleus | 0.01 | -0.059 | 0.039 | 0.127 | 0.326 |
| Medial.nucleus | 0.1 | -0.042 | 0.039 | 0.283 | 0.483 |
| Medial.nucleus | 1 | -0.064 | 0.038 | 0.092 | 0.280 |
| Cortical.nucleus | 1.00E-08 | 0.014 | 0.036 | 0.700 | 0.826 |
| Cortical.nucleus | 1.00E-07 | 0.015 | 0.035 | 0.659 | 0.800 |
| Cortical.nucleus | 1.00E-06 | 0.017 | 0.035 | 0.622 | 0.769 |
| Cortical.nucleus | 1.00E-05 | -0.011 | 0.035 | 0.762 | 0.864 |
| Cortical.nucleus | 1.00E-04 | -0.013 | 0.037 | 0.723 | 0.838 |
| Cortical.nucleus | 0.001 | -0.035 | 0.037 | 0.339 | 0.536 |
| Cortical.nucleus | 0.01 | -0.043 | 0.037 | 0.242 | 0.456 |
| Cortical.nucleus | 0.1 | -0.046 | 0.037 | 0.213 | 0.433 |
| Cortical.nucleus | 1 | -0.070 | 0.036 | 0.052 | 0.222 |
| Corticoamygdaloid.transitio | 1.00E-08 | -0.026 | 0.032 | 0.419 | 0.601 |
| Corticoamygdaloid.transitio | 1.00E-07 | -0.035 | 0.031 | 0.264 | 0.481 |
| Corticoamygdaloid.transitio | 1.00E-06 | -0.037 | 0.031 | 0.230 | 0.447 |
| Corticoamygdaloid.transitio | 1.00E-05 | -0.068 | 0.031 | 0.029 | 0.170 |
| Corticoamygdaloid.transitio | 1.00E-04 | -0.087 | 0.032 | 0.007 | 0.087 |
| Corticoamygdaloid.transitio | 0.001 | -0.063 | 0.032 | 0.053 | 0.222 |
| Corticoamygdaloid.transitio | 0.01 | -0.046 | 0.032 | 0.156 | 0.364 |
| Corticoamygdaloid.transitio | 0.1 | -0.023 | 0.033 | 0.490 | 0.659 |
| Corticoamygdaloid.transitio | 1 | -0.017 | 0.032 | 0.601 | 0.756 |
| Paralaminar.nucleus | 1.00E-08 | -0.021 | 0.027 | 0.428 | 0.607 |
| Paralaminar.nucleus | 1.00E-07 | -0.031 | 0.026 | 0.227 | 0.443 |
| Paralaminar.nucleus | 1.00E-06 | -0.038 | 0.026 | 0.148 | 0.353 |
| Paralaminar.nucleus | 1.00E-05 | -0.063 | 0.026 | 0.015 | 0.120 |
| Paralaminar.nucleus | 1.00E-04 | -0.076 | 0.027 | 0.006 | 0.072 |
| Paralaminar.nucleus | 0.001 | -0.050 | 0.027 | 0.070 | 0.262 |
| Paralaminar.nucleus | 0.01 | -0.047 | 0.027 | 0.085 | 0.276 |
| Paralaminar.nucleus | 0.1 | -0.006 | 0.028 | 0.818 | 0.886 |
| Paralaminar.nucleus | 1 | -0.007 | 0.027 | 0.788 | 0.875 |
| Whole_amygdala | 1.00E-08 | -0.030 | 0.027 | 0.266 | 0.481 |
| Whole_amygdala | 1.00E-07 | -0.039 | 0.026 | 0.139 | 0.346 |
| Whole_amygdala | 1.00E-06 | -0.045 | 0.026 | 0.086 | 0.276 |
| Whole_amygdala | 1.00E-05 | -0.073 | 0.026 | 0.005 | 0.070 |
| Whole_amygdala | 1.00E-04 | -0.070 | 0.027 | 0.011 | 0.097 |
| Whole_amygdala | 0.001 | -0.060 | 0.027 | 0.028 | 0.169 |
| Whole_amygdala | 0.01 | -0.051 | 0.028 | 0.062 | 0.245 |
| Whole_amygdala | 0.1 | -0.021 | 0.028 | 0.444 | 0.625 |
| Whole_amygdala | 1 | -0.029 | 0.027 | 0.286 | 0.483 |

**Supplementary Table 4. *APOE* ε4 status ~ Subregions**

| ROI | BETA | SE | P | FDR |
| --- | --- | --- | --- | --- |
| Hippocampal_tail | 0.107 | 0.084 | 0.202 | 0.989 |
| subiculum | 0.037 | 0.076 | 0.626 | 0.989 |
| CA1 | 0.070 | 0.075 | 0.351 | 0.989 |
| hippocampal.fissure | 0.031 | 0.075 | 0.673 | 0.989 |
| presubiculum | -0.047 | 0.073 | 0.520 | 0.989 |
| parasubiculum | 0.008 | 0.076 | 0.919 | 0.989 |
| molecular_layer_HP | -0.002 | 0.078 | 0.981 | 0.989 |
| GC.ML.DG | -0.005 | 0.074 | 0.943 | 0.989 |
| CA3 | 0.082 | 0.078 | 0.294 | 0.989 |
| CA4 | 0.009 | 0.074 | 0.899 | 0.989 |
| fimbria | 0.002 | 0.072 | 0.975 | 0.989 |
| HATA | 0.049 | 0.079 | 0.533 | 0.989 |
| Whole_hippocampus | 0.043 | 0.071 | 0.544 | 0.989 |
| Lateral.nucleus | -0.005 | 0.066 | 0.937 | 0.989 |
| Basal.nucleus | 0.017 | 0.069 | 0.801 | 0.989 |
| Accessory.Basal.nucleus | 0.046 | 0.075 | 0.543 | 0.989 |
| Anterior.amygdaloid.area.AAA | 0.098 | 0.073 | 0.175 | 0.989 |
| Central.nucleus | 0.065 | 0.082 | 0.423 | 0.989 |
| Medial.nucleus | -0.027 | 0.087 | 0.759 | 0.989 |
| Cortical.nucleus | 0.049 | 0.082 | 0.556 | 0.989 |
| Corticoamygdaloid.transitio | 0.039 | 0.077 | 0.611 | 0.989 |
| Paralaminar.nucleus | -0.001 | 0.068 | 0.989 | 0.989 |
| Whole_amygdala | 0.024 | 0.069 | 0.726 | 0.989 |
